# Alginate impressions: A practical perspective

**DOI:** 10.4103/0972-0707.43416

**Published:** 2008

**Authors:** V Vidyashree Nandini, K Vijay Venkatesh, K Chandrasekharan Nair

**Affiliations:** Department of Prosthodontics & Implantology, Meenakshi Ammal Dental College, Chennai, India; 1 Department of Conservative Dentistry & Endodontics, Meenakshi Ammal Dental College, Chennai, India

**Keywords:** Alginate, impressions, irreversible hydrocolloid

## Abstract

The choice of an impression material for a particular situation depends on the treatment being provided, operator preference, and so on. Even with the introduction of more advanced and more accurate rubber base impression materials, irreversible hydrocolloid impression materials have stood the test of time. This article gives a detailed perspective of how best to make alginate impressions.

Alginate is an elastic, irreversible hydrocolloid impression material. Irreversible hydrocolloid impressions form an inseparable part of indirect restorations. Alginate is one of the most frequently used dental materials; and alginate impression is a simple, cost-effective, and indispensable part of dental practice. Yet very few people can make alginate impressions just right the first time. For many years, alginate impression material has been a staple of most dental practices. They form a major bulk of our clinical practice even today; therefore, it becomes mandatory to understand the material and follow certain fundamental guidelines for flawless, predictable impressions and hence avoid repeat impression/restorations. The purpose of this article is to provide clinical do's and don'ts while using alginate material for impressions.

Irreversible hydrocolloid can be used in preliminary impressions, provisional crown-and-bridge impressions, study models, opposing dentition impressions. Alginates are used for impressions in orthodontic models, sports mouth guards, and bleaching trays; and more.[1] A study has shown that alginate impressions can be used as final impressions for indirect restorations when the preparation margins are chamfer.[2]

## CHEMISTRY AND SETTING

The powder contains sodium alginate, calcium sulfate, trisodium phosphate, diatomaceous earth, zinc oxide, and potassium titanium fluoride. On mixing the powder with water, a sol is formed, a chemical reaction takes place, and a gel is formed. Here, sodium alginate reacts with calcium sulfate, resulting in sodium sulfate and calcium alginate. This reaction occurs too quickly often during mixing or loading of the impression tray. Hence it is slowed down by the addition of trisodium phosphate to the powder. Trisodium phosphate reacts with calcium sulfate to produce calcium phosphate, preventing calcium sulfate from reacting with sodium alginate to form a gel. This second reaction occurs in preference to the first reaction until the trisodium phosphate is used up, and then alginate sets as a gel. There is a well-defined working time during which there is no viscosity change.[3]

Alginate materials possess the qualities of good surface detail and faster reaction at higher temperatures. They are elastic enough to be drawn over the undercuts but tear over deep undercuts and are not dimensionally stable on storing due to evaporation. Alginates are nontoxic and nonirritant. Alginate powder is unstable on storage in the presence of moisture or in warm temperatures. Alginate impression materials are hydrophilic in nature, and this property facilitates making of accurate impressions in the presence of saliva or blood.[4] It has a low wetting angle and hence full arch impressions are easily captured. Impressions made with irreversible hydrocolloids are easier to remove than those with elastomeric materials. As their tear strength is low, they can reproduce subgingival contours and anatomy but tear upon removal.[5] They are good for only one pour per impression.[6]

Alginate impression materials are easy to use, less expensive, with quick setting time. The setting time can be controlled with the temperature of water used. They are mildly flavored. Their disadvantages include less accurate reproduction of details as compared with elastomeric impression materials, poor dimensional stability, and that they are messy to work with.

The most popular form of alginate is supplied as a powder, which is mixed with water. Many alginates are supplied with a reaction indicator that changes color of the impression when the material is set; and presently, dustless alginates are preferred. Powder may be available in bulk form in containers or in individual sealed pouches. Paste type of alginate is also available. Paste form is available in two viscosities, tray and syringe viscosities. The paste-type material has a shorter gelation time than the powder-type material. The best surface quality can be obtained with the paste-type material. Studies suggest that a paste-type material would better meet the requirements of an alginate impression material.[7]

Christensen GJ[8] had observed that high points on indirect restorations are a result of inaccurate opposing arch impressions from alginate materials. Predictable alginate impressions can be made by adhering to a protocol. The steps involved are selection of impression tray, mixing and loading of alginate impression material, preparing the mouth, making the impression, removal/inspection of the impression, and storage and disinfection.

## SELECTION OF IMPRESSION TRAY

As is the case with all impression materials, it is important to select the correct tray for the dental arch. The stock trays to be selected for alginate should be perforated. Alginate adhesives can be used, apart from perforations, for retention of alginate to the impression tray. Use of alginate adhesives overcomes displacing forces during withdrawal of the impression from the mouth. Alginate adhesives are available as paint-on or spray-on. The use of brush can result in cross-infection of patients. After application of an alginate adhesive, it is allowed to dry for 5 minutes.

More frequently, the stock trays would require some customization in the form of tray modification. Modifications can be done with wax, tracing stick impression compound, or heavy-bodied silicone, depending on the operator's convenience. Stick compound is preferable as wax is nonrigid. Impression trays and their modifications should be rigid. Modified impression tray must be placed in the mouth and muscle trimmed.

Before making an impression, the dental arch to be impressed should be examined carefully. If there are any existing fixed partial dentures, the pontics require to be blocked out with wax so that the impression does not tear in these regions.

**Figure 1:**
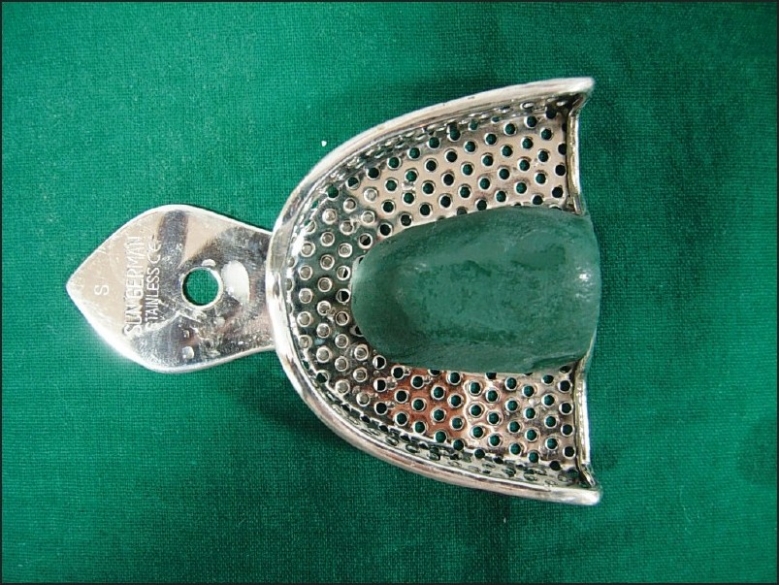
Modification of impression tray for high palatal vault

**Figure 2:**
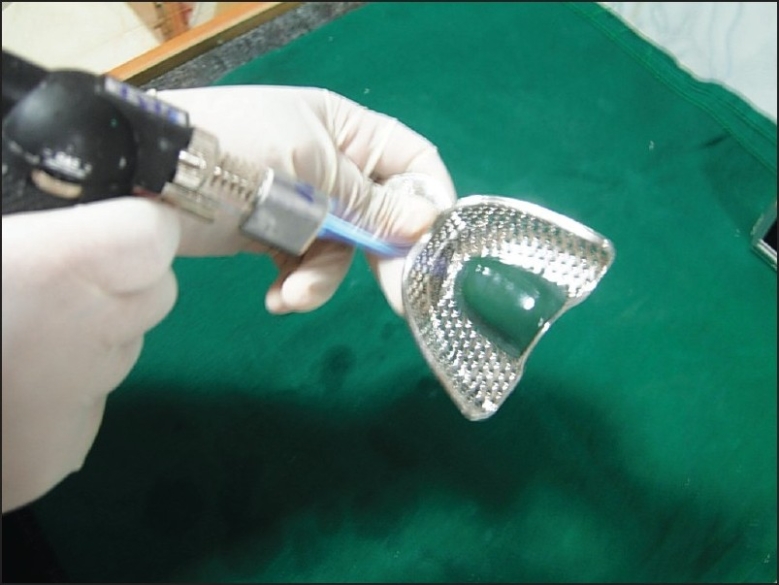
Softening the tracing stick compound before placing cotton

**Figure 3:**
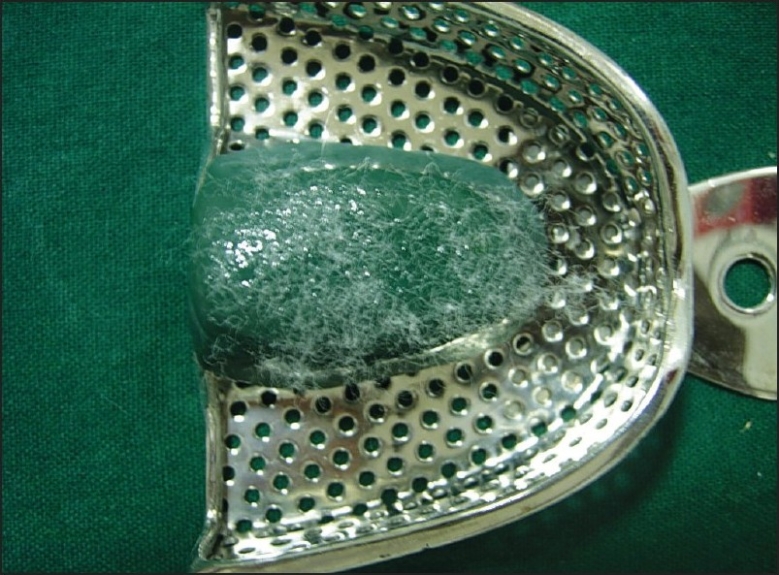
Embedded cotton fibers, provide retention with alginate

If the patient has a high palatal vault, tracing stick compound can be used in the center of the maxillary tray to reduce the bulk of alginate impression material [Figure 1]. In case teeth are missing in one posterior quadrant, modeling compound is softened in a water bath and placed in the tray to make an impression of the edentulous areas. The imprints of the teeth should be cut out with a sharp knife so that the compound has definite stops against the maxillary tuberosity (ensures teeth do not touch the tray and distort impression). Compound is trimmed so that 3 to 5 mm of clearance between compound and mucosa exists except in posterior palatal seal area. This compound modification of the impression tray should be firmly adhering to the tray. If not, wax spatula is heated to merge with the tray borders carefully. The compound surface is softened with a flame, and a ball of cotton is pressed into the surface for a few seconds, leaving short cotton fibers embedded [Figures 2, and 3]. The cotton fibers provide retention for alginate material. Impression tray is placed in cold water to harden the compound.

Presently we have triple trays for full arch alginate impressions (Alfa Tray, Premier®, U.S.A.). They are disposable, avoiding cross-contamination. They are palate-free trays and hence restrict overflow of material and eliminate gagging.

## MIXING AND LOADING ALGINATE

**Figure 4:**
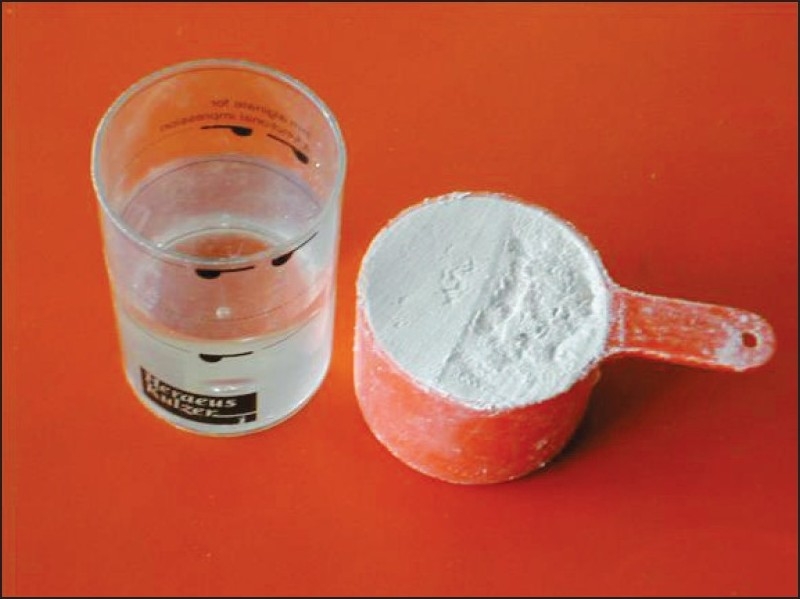
Measuring cylinder, scoop

**Figure 5:**
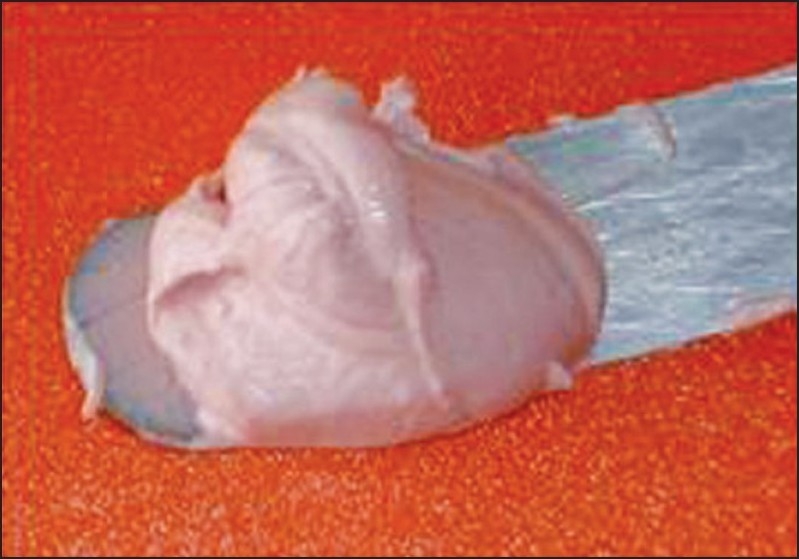
A smooth, creamy mix

Commonly used alginate materials are supplied in containers. A scoop is provided for measuring the powder; and a cylindrical plastic measuring cylinder, for measuring the water proportion [Figure 4]. Some water supplies contain large amounts of minerals that can affect the accuracy and setting time of the alginate. In such cases, distilled or demineralized water can be used.[9] Mixing is initiated by adding measured quantity of water to clean flexible rubber bowl. This is followed by the addition of correctly proportioned powder. Colder water can be used if longer working time is desired. Setting time should be controlled by varying water temperature, and not the consistency of mix. Mixing should be rapid with a wide-bladed spatula. The resultant mix should be creamy in consistency but must not drip off the spatula when lifted from the bowl [Figure 5].

Mechanical mixing of alginate in devices such as the Alginator II (Dux Dental, Oxnard, California) or the Combination Unit (Whip Mix, Louisville, Ky.) ensures that the alginate mix is the same each time they are mixed. Mixing time is 60 seconds for hand spatulation and 15 seconds for mechanical.

The required amount of material is loaded onto the tray. The tray must be filled with the impression material up to the tray borders and any excess unsupported material (over-filled tray) at the periphery must be removed with the mixing spatula. The surface of the alginate is smoothed with a wet gloved finger.[1]

## PREPARING THE MOUTH BEFORE IMPRESSIONS

For dentate cases, occlusal surfaces of teeth should be blown off with an air syringe to remove debris and saliva, in order to minimize air-blows. At the same time, the teeth should not be left to dry completely since alginate material sticks to dried teeth as the thin film overlying the teeth is removed. When the surfaces are dry, the alginate radicals in the impression material form chemical bonds with hydroxyapatite crystals of the enamel; hence alginate tears upon removal.[9] Having the patient rinse with water and mouthwash mixture will eliminate mucin and lower the surface tension, thereby eliminating air bubbles. If repetitive impressions are made with alginate, the film over the teeth is lost and getting a satisfactory impression is prevented. While repeating impressions, the patient must be asked to rinse the mouth to re-hydrate and produce a new film over the teeth for accurate impressions

For removable prostheses, impressions of the sulci are very essential. Hence pre-packing of the sulci, especially lower lingual, upper labial, hamular notch/distobuccal areas, should be considered.

## IMPRESSION MAKING

The mixed alginate should be rubbed onto the occlusal surfaces with a gloved finger to fill the occlusal grooves, allowing accurate reproduction of the occlusal tooth anatomy. Some alginate must be placed in the palatal vault. Impression tray is positioned in the mouth by retracting the patient's lips on one side with a mouth mirror/gloved finger; and on the other side, by rotating the tray into the mouth. The tray has to be centered in position in the mouth; and with light pressure, impression is held in place. The soft tissues, especially labial flange, should be relieved and manipulated for the alginate to flow into the sulci and record the details.

When tray is seated, pressure should be released immediately and the tray should be held lightly in place to prevent unseating. It is imperative to release pressure as soon as the tray is seated. Alginate materials start setting from the tooth surface to the impression tray. Pressure will cause impression to set under strain. On removing the impression from mouth, these strains will be released, causing distortion and an inaccurate cast. Moving the impression tray during gelation will incorporate similar strains.

## REMOVAL AND INSPECTION OF IMPRESSION

Once set, the impression has to be removed with a firm, quick snap. The impression should not be rocked or twisted before or during removal of the impression. This is to minimize the time for which the set material is distorted as it moves over the teeth. The seal between tissues and the impression may have to be removed before removal of maxillary impressions, by gently pushing with the gloved finger or by using air-syringe into the buccal sulcus. During removal of the maxillary impression, the operator's index fingers (of both the hands) should be in the buccal sulci to break the seal while thumb holds the tray handle and the other fingers support the impression tray.

Upon removal of the impression from the mouth, impression is inspected for defects under good lighting before it is rinsed. The impression should be rinsed with cold water to remove any saliva or blood. Most patients have thin, serous saliva. This type of saliva can be removed by holding the impression under gently running cool tap water. But thick, ropy saliva is difficult to remove. A thin layer of dental stone powder can be sprinkled onto the surface of the impression. The stone adheres to saliva and acts as a disclosing agent. When impression is placed under running tap water, the saliva will be seen and can be removed by light brushing with wet camel's hair brush.[10] The impression should then be covered in a damp gauze/napkin to prevent syneresis (generally, not recommended to place in water, which would cause imbibition-expansion).

Excess unsupported alginate should be removed with a sharp knife. If the tray is left on a firm surface with unsupported material, the impression would distort as the weight of the impression acts directly on the unsupported material. This occurs in the posterior areas of the upper and lower impressions, and it will lead to anteroposterior distortion of the cast.

## STORAGE AND DISINFECTION

Set alginate undergoes imbibition and syneresis if left in a normal clinical environment. The time before cast-pouring is critical. After being removed from the mouth, alginate impressions should be washed with a water spray, disinfected by means of the practitioner's choice of disinfection procedures, and dried until the shine just disappears. The impression has to be covered with damp gauze and left in a zip-lock plastic bag until the cast is poured.[1] Cast-pouring must be delayed for 10 minutes, as the elastic recovery of alginate impression material is slow.

Distortion can be a problem if disinfection guidelines are not strictly adhered to. Hydrocolloids are hydrophilic in nature; hence they swell if immersed in water or disinfectant. Disinfectant sprays are used for alginate impressions, but they do result in air bubbles in the cast. Immersion disinfectants like 1% sodium hypochlorite or 2% glutaraldehyde can result in changes of 0.1%, and hence the quality of the impression surface may not be impaired if the recommended period of time is strictly followed.[9]

## CAST FABRICATION

Alginate impressions should be poured using vacuum-mixed stone and vibrator. A thick mix can trap air bubbles. The stone should be allowed to set in trays with the teeth down. If tray is turned upside down onto base of stone, there would be a tendency for water to rise to the highest point (cusp tips). This can result in faulty, very soft cusps on the model. Inverting the tray may also “bend” alginate away from tray if excess material has not been trimmed away prior to pouring. Cast has to be removed immediately after adequate set; otherwise, the model would have “moth-eaten” appearance.

## CONCLUSION

Making impressions is an important part of indirect restorations that is often overlooked. Soon we would be stepping into the era of digital impressions. They can provide unmatched precision, making repeat impressions a thing of the past. Till such time, reliable alginate impressions can be made by following an appropriate technique. The factors that can diminish the accuracy and reliability can be predictably and easily avoided with an understanding of the clinical situation, knowledge of the recent advances, use of skillful technique, and careful observation by both the clinician and the dental technician.
